# A Clinicopathological Study of Early-Stage Synchronous Bilateral Breast Cancer: A Retrospective Evaluation and Prospective Validation of Potential Risk Factors

**DOI:** 10.1371/journal.pone.0095185

**Published:** 2014-04-15

**Authors:** Jia-jian Chen, Yan Wang, Jing-yan Xue, Ying Chen, Ya-ling Chen, Qin Xiao, Wen-tao Yang, Zhi-min Shao, Jiong Wu

**Affiliations:** 1 Department of Breast Surgery, Fudan University Shanghai Cancer Center; Department of Oncology, Shanghai Medical College, Fudan University, Shanghai, China; 2 Department of Ultrasound, Fudan University Shanghai Cancer Center; Department of Oncology, Shanghai Medical College, Fudan University, Shanghai, China; 3 Department of Diagnostic Radiology, Fudan University Shanghai Cancer Center; Department of Oncology, Shanghai Medical College, Fudan University, Shanghai, China; 4 Department of Pathology, Fudan University Shanghai Cancer Center; Department of Oncology, Shanghai Medical College, Fudan University, Shanghai, China; University of North Carolina School of Medicine, United States of America

## Abstract

**Background:**

The aim of the present study was to investigate potential risk factors for synchronous bilateral breast cancer sBBC).

**Methods:**

A retrospective analysis was performed of patients diagnosed and treated with operable bilateral breast cancer (BBC) between June 2007 and December 2011. Risk factors for sBBC were evaluated in this cohort and further validated in a prospective observational validation analysis of patients between January 2012 and December 2012. Patients treated with operable unilateral breast cancer during the same period were used as a control group.

**Results:**

A total of 11,247 patients with primary breast cancer underwent operations at the Fudan University Shanghai Cancer Center between June 2007 and December 2012. The incidence of sBBC was 1.6%. The age at diagnosis (HR = 2.4, 95% C.I.: 1.4–4.0, p = 0.001), presence of sclerosing adenosis (HR = 11.8, 95% C.I.: 5.3–26.3, p<0.001), lobular carcinoma component involvement (HR = 5.6, 95% C.I.: 2.6–12.1, p<0.001), and family history of first-degree relatives with breast cancer (HR = 2.0, 95% C.I.: 1.1–3.4, p<0.001) were independent risk factors for sBBC. A subsequent validation study failed to confirm the significance of family history. No significant difference on survival was found between patients with early-stage sBBC and control cases.

**Conclusions:**

Patients with the presence of sclerosing in the affected breast, and lobular carcinoma component involvement may be at high risk for developing sBBC. This study supports the hypothesis that the host-carcinoma biological relationship, especially for the tumor microenvironment, played a critical role in the carcinogenesis of sBBC.

## Introduction

There is an increasing number of women who are at risk for developing synchronous bilateral breast cancer (sBBC), owing to the increasing morbidity,improved diagnostic technologies and management strategies. The first description of sBBC was published by Kilgore in 1921, who defined sBBC based on the simultaneous diagnosis of tumors in both breasts [1]. In later publications, a time interval between the diagnoses of the tumors was introduced. However, the length of the time interval widely varies among different retrospective studies, i.e., from one month [2] to five years [3]. The definition of synchronous and metachronous bilateral breast cancer (mBBC) according to length of the time and the clinical value of this classification remains controversial.

Despite recent ongoing studies, the epidemiology and impact on the survival of patients with sBBC was still under debate. Several clinicopathological parameters such as age at diagnosis [4]–[6], histopathological type [5], [7]–[9], family history [9], [10], and hormone receptor status [11] have been considered as important risk factors for developing bilateral carcinomas. However, several flaws in previous studies might significantly affect the results on which these conclusions are based. First, most of these studies recruited patients over a long time period, i.e., greater than ten years, which might have resulted in bias due to differences in diagnostic technologies and management strategies. Second, few studies eliminated patients with stage IIIb or IIIc (T_4_ or N_3_) and even stage IV disease. It is difficult to distinguish primary bilateral disease and metastatic disease when the disease has progressed to such stages, which might introduce bias in the selection of patients. Finally, several studies [12] excluded patients with in situ carcinoma from analysis, which remains a topic of debate.

This study aimed to investigate the potential risk factors for sBBC in a retrospective series of patients within a short study interval, followed by validation in a prospective patient series.

## Materials and Methods

### Patients

Patients diagnosed and treated with operable BBC in the Fudan University Shanghai Cancer Center between June 2007 and December 2011 were enrolled in the retrospective analysis. Risk factors for sBBC were evaluated in this cohort. To validate the risk factors, we performed a prospective observational analysis of patients with sBBC between January 2012 and December 2012. Patients treated with operable unilateral breast cancer (UBC) during the same period were used as a control group. To avoid the risk of misclassifying metastatic disease, patients with stage IIIb or IIIc (T_4_ or N_3_) or IV disease were excluded from the risk factor-related analysis.

The study protocol was approved by the Fudan University Shanghai Cancer Center ethics committee. The clinicopathological and epidemiological parameters of each patient were recorded in the electronic medical records system at the Fudan University Shanghai Cancer Center, and was anonymized and de-identified prior to analysis. The seventh edition of the American Joint Committee on Cancer classification system was used for staging.

### Statistical Analysis

An independent samples *t* test and Kruskal-Wallis test were performed to compare continuous variables, while Fisher’s exact test was used to analyze categorical variables. Multiple regression analysis was used to determine independent risk factors for bilateral breast cancer. Survival distributions were analyzed using the Kaplan-Meier method. All tests with *p*<0.05 were considered indicative of statistical significance (SPSS statistical analysis program, version 20.0; SPSS Inc., Chicago, IL, USA).

## Results

Between June 2007 and December 2012, a total of 11,247 patients with primary breast cancer underwent operations at the Fudan University Shanghai Cancer Center. Among these patients, final pathology confirmed T_4_ or N_3_ disease in 1,121 patients with UBC and 20 patients with BBC; these patients were thus excluded from the risk factors-related analysis.

### Distribution and Definition of Patients with BBC

**Figure 1:**
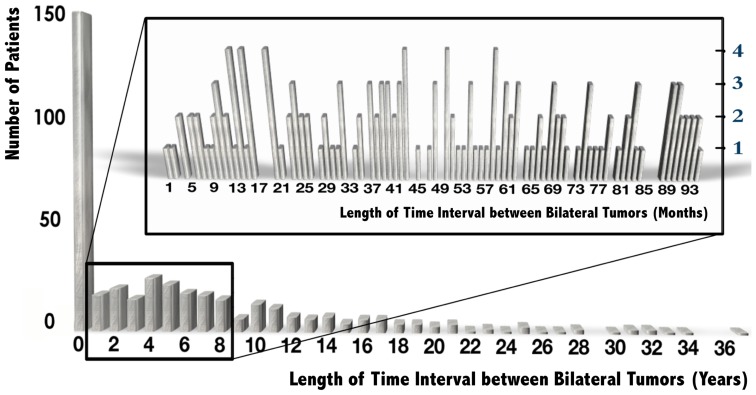
Distribution of patients with BBC according to the time interval between bilateral tumors between June 2007 and December 2012. The distribution in the first 8 years is shown by month.

The length of time between the first and second primary carcinomas varied widely among the 396 patients with early-stage bilateral carcinoma from the same time period, to over 36 years. A total of 149 (37.6%) patients with BBC were diagnosed within 10 workdays. Although the incidence of BBC continued to reduce with the length of time from surgery, the trend in the incidence of BBC did not change significantly during the first 8 years after surgery [Figure 1]. For the definition of sBBC and mBBC, the length of the time interval (1 month, 3 months, 6 months, or 1 year) did not significantly affect the results of the statistical analysis.

In this study, a time interval of 1 year between bilateral carcinomas was utilized because many patients may need adjuvant therapy for almost 6 months and an additional 6 months of follow-up to avoid the misdiagnosis of an existing contralateral carcinoma. According to this classification, 161 (1.6%) patients were classified with sBBC, and 235 (2.3%) patients had mBBC.

**Figure 2:**
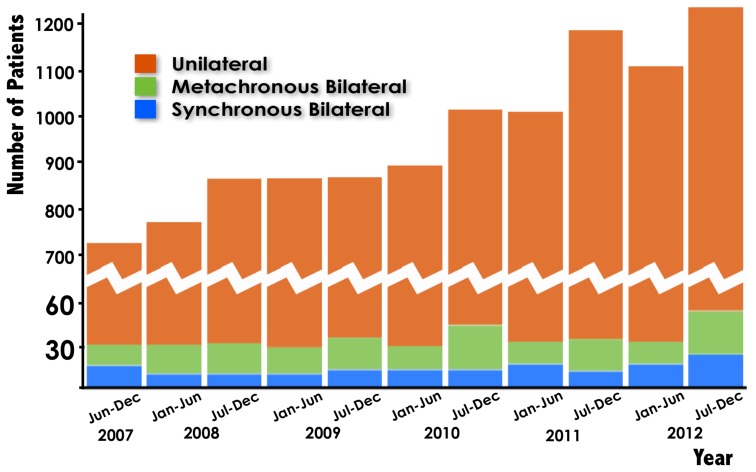
The incidence of sBBC and mBBC compared with that of UBC among patients diagnosed between June 2007 and December 2012.

In addition, the incidence of sBBC and mBBC remained constant for all years, while that of UBC significantly increased over the years [Figure 2].

### Evaluation of Possible Risk Factors for sBBC

A retrospective analysis was performed of 117 patients with sBBC and 7,400 patients with UBC diagnosed between June 2007 and December 2011. The demographic and clinicopathological characteristics of the patients are summarized in Table 1.

**Table 1:** Patient demographic and clinicopathological characteristics.

Variables	sBBC (n = 117)	UBC (n = 7400)	p value
**Age (median, range)**	53 (30–89)	51 (17–98)	<0.001
**Family history**	16	482	0.010
**Histopathology** *			<0.001
** Ductal**	98	6411	
** Lobular**	8	79	
** Others**	11	387	
**Stage**			0.360
** 0–I**	41	2562	
** II**	68	3619	
** IIIa**	8	696	
**ER positive** **	102/116	3202/4365	0.188
c-erbB-2 **positive** **	48/111	1729/4306	0.658
**Accompanying benign diseases**			
** Adenoma**	3	89	0.204
** Papilloma**	2	73	0.361
** Sclerosing Adenosis** ***	8	45	<0.001

sBBC: synchronous bilateral breast cancer; UBC: unilateral breast cancer.

*Carcinomas with lobular carcinoma component involvement in either breast were classified as ***Lobular***, while those with the involvement of other carcinoma components, with the exception of ductal or lobular carcinoma, in either breast were classified as ***Other***.

**ER or c-erbB-2 expression in either breast was considered positive.

***Sclerosing adenosis was a major component, including carcinomas arising from sclerosing adenosis and carcinomas involved with sclerosing adenosis.

Compared with patients with UBC, those with sBBC were generally older (median age: 53 vs. 51 years, p<0.001), and no patients were diagnosed with sBBC before age 30. Patients older than age 45 had a 2.4-fold higher risk of sBBC compared with those younger than age 45, according to a multiple regression analysis [Table 2]. Patients with family history of first-degree relatives with breast cancer or those with lobular carcinoma component involvement also tended to develop sBBC, which were also confirmed by the subsequent multiple regression analysis [Table 2]. However, no significant difference was found in the distribution of UICC stage and ER and c-erbB-2 positivity between patients with sBBC and those with UBC.

**Table 2:** Multiple regression analysis of risk factors for sBBC in the retrospective study.

Risk factors	HR	95% C.I.	P
**Age (<45 yr, ≥45 yr)**	2.4	1.44–3.97	0.001
**Sclerosing Adenosis**	11.8	5.3–26.3	<0.001
**Lobular Carcinoma**	5.6	2.6–12.1	<0.001
**Family History**	2.0	1.1–3.4	0.018

To ensure the integrity of our research, the details of accompanying benign diseases in the affected breasts were recorded in the analysis. Unexpectedly, we discovered that the percentage of patients with an accompanying sclerosing adenosis in the affected breast, including carcinomas arising from or involved with sclerosing adenosis, was significantly higher in patients with sBBC than in patients with UBC (6.8 vs. 0.6%, p<0.001). Patients with accompanying sclerosing adenosis had a strong trend toward developing sBBC [Table 2].

### Validation of Risk Factors for sBBC

Since the numbers of patients with UBC and sBBC in the retrospective study varied widely, to validate the results of the retrospective study, we conducted a prospective observational study from January 2012 to December 2012. During this time period, a total of 2,310 patients with UBC and 44 patients with sBBC were diagnosed and treated in our institution.

Patients with sBBC were significantly older than those with UBC (median age: 50 vs. 53 years, p<0.001). The validation study also confirmed lobular carcinoma component involvement as a significant risk factor for sBBC (sBBC: UBC = 9.1%∶2.0%, p = 0.016). However, the percentage of patients with a family history of first-degree relatives with breast cancer was similar for patients with sBBC and those with UBC (sBBC:UBC = 13.6%∶7.2%, p = 0.151), indicating that family history was less significant than the presence of sclerosing adenosis or a lobular carcinoma component for predicting the risk of sBBC.

Based on the results of the retrospective study, the pathologists in our institute also focused more on the existence of sclerosing adenosis in breast specimens. The diagnosis of accompanying sclerosing adenosis in patients with breast cancer was more frequent in 2012 than previously (4.0 vs. 0.7%, p<0.001). The presence of accompanying sclerosing adenosis as a major component was also confirmed to be an important risk factor for sBBC (sBBC:UBC = 13.6%∶3.8%, p = 0.011).

### Clinicopathological Characteristics of Different Risk Factors Related to sBBC

Among the patients with sBBC diagnosed and treated between June 2007 and December 2012, a total of 39 (24.2%) patients had at least one of the risk factors mentioned in Table 2, which includes the presence of sclerosing adenosis, lobular carcinoma involvement, or a family history of first-degree relatives with breast cancer.

Patients with sclerosing adenosis-related sBBC were the youngest group among all patients with sBBC [Table 3]. In addition, up to 64.3% of the patients with sclerosing adenosis-related sBBC had in situ carcinoma with or without microinvasion in both breasts, while the proportion of such patients with lobular carcinoma- and family history-related sBBC was less than 10%; the difference among groups reached statistical significance (p = 0.009). However, the proportion of node-positive disease in patients with invasive carcinoma was similar among groups. All patients with sclerosing adenosis- or lobular carcinoma-related sBBC had hormone receptor-positive disease in at least one breast. Although the distribution of molecular subtypes was similar among different risk factors related to sBBC, we still observed some trends. A total of 85% of the patients with sclerosing adenosis-related sBBC and 96% of the patients with lobular carcinoma-related sBBC were of the luminal subtype. Approximately one-fourth of the family history-related sBBCs were of the c-erbB-2-positive and triple-negative subtypes.

**Table 3:** Clinicopathological characteristics of different risk factors related to sBBC.

Variable	Sclerosing adenosisrelated(n = 14)	Lobular carcinomarelated(n = 12)	Family historyrelated(n = 21)	Others(n = 122)	P
**Age**	48.8 (42.5–65.3)	50.4 (39.4–59.1)	53.0 (36.1–73.4)	55.1 (30.1–88.8)	0.026
**in situ** **Carcinoma (±mi)** *	9 (64.3%)	1 (8.3%)	2 (9.5%)	19 (15.6%)	0.009
**Node+ in** **invasive carcinoma**	2 (40.0%)	4 (36.4%)	9 (47.4%)	40 (38.8%)	0.973
**HR positive** **	14 (100%)	12 (100%)	17 (81.0%)	105 (86.8%)	0.959
**Molecular** **subtype** ***					0.423
**Luminal A**	13 (46.4%)	18 (75.0%)	22 (52.4%)	137 (56.1%)	
**Luminal B**	11 (39.3%)	5 (20.8%)	9 (21.4%)	54 (22.1%)	
c-erbB-2 **Positive**	3 (10.7%)	1 (4.2%)	6 (14.3%)	27 (11.1%)	
**Triple Negative**	1 (3.8%)	0	4 (9.5%)	17 (7.0%)	

±mi: with or without microinvasion; HR: hormone receptor.

*Patients with in situ carcinoma with or without microinvasion in both breasts.

**Patients with hormone receptor-positive disease in either breast were considered positive.

***Patients with BBC were considered to have two independent carcinomas in this analysis.

### Treatment and Survival of Patients with sBBC

All patients received standardized multi-disciplinary therapies according to the guidelines of our institution. No statistical significance was found for the proportion of patients who received adjuvant chemotherapy and adjuvant endocrine therapy. However, patients with sBBC were significantly more likely to undergo bilateral mastectomy than patients with UBC (90.1 vs. 65.3%, p<0.001). Similarly, approximately 40.4% of the patients received bilateral axillary lymph node dissection. Sentinel lymph node biopsy for both axilla was performed in 27.3% of patients. With a median follow-up period of 37 months, no significant difference was found between the patients with early-stage sBBC and those with early-stage UBC in breast cancer-specific disease-free survival (95.0 vs. 95.8%, p = 0.261). However, among the patients with T_4_ or N_3_ disease, the breast cancer-specific disease-free survival was merely 50.0%, with a median follow-up of 18 months.

## Discussion

This study investigated potential risk factors for sBBC in a retrospective series of patients diagnosed and treated in a recent four-year period, followed by validation of the identified risk factors in a prospective series of patients. Although the time period may result in a relatively short period of follow-up for the survival-related analysis, it has the significant advantage of eliminating bias due to differences in the diagnostic technologies and management strategies used across years, thus ensuring the accuracy and credibility of the results of the risk factor analysis. Another major advantage of this study is that it is the first to consider accompanying benign disease in the breast to determine the potential effects of benign lesions or microenvironment components on the carcinogenesis of BBC.

### Definition of Primary sBBC

The reported incidence of sBBC has remained stable at approximately 2% of all breast cancer cases, ranging from 0.7 [12] to 3.2% [13], in publications from the past ten years and was 1.6% in this study. The diagnosis of sBBC is based on two aspects: the differential diagnosis of metastatic disease and the length of the time interval between bilateral carcinomas.

To distinguish between BBC and metastatic disease in the contralateral breast, many studies [10], [12], [14]–[16] refer to the criteria described by Chaudary et al. [17] in 1984, which include the demonstration of in situ disease, different histological types, a greater degree of histological differentiation, and no evidence of local, regional, or distant metastasis. However, a large number of patients with BBC diagnosed within a relatively short interval have the same histological type and differentiation grade in bilateral carcinomas. The second and third Chaudary criteria may be omitted for the diagnosis of sBBC. Whether BBC patients with stage IIIb or IIIc (T_4_ or N_3_) disease in either breast should be excluded from the diagnosis of sBBC is also worthy of discussion. It is difficult to distinguish primary bilateral and metastatic disease when it has progressed to such stages, which might introduce bias in patient selection. Several studies have suggested worse survival for sBBC compared with UBC; however, a significantly higher proportion of local advanced disease was found in patients with sBBC compared with those with UBC [5], [10], [12], [15]. The breast cancer-specific disease-free survival was merely 50.0% with a median follow-up of 18 months for BBC patients with stage IIIb or IIIc disease in either breast in this study. The poorer survival of such bilateral carcinomas could not be directly attributed to the occurrence of bilateral carcinoma. It is more likely that a portion of patients with locally advanced and contralateral metastatic disease were misclassified as having sBBC, resulting in bias in the survival analysis, as supported by a study by Nichol et al. [18], in which the overall 10-year breast cancer-specific survival was significantly higher for UBC than sBBC cases and was equal after matching for risk.

The length of the time interval between bilateral carcinomas is the other aspect of the diagnosis of sBBC. This study indicated that 37.6% of patients with BBC were diagnosed within 10 workdays and that the incidence of BBC cancer remained stable over the subsequent 8 years [Figure 1]. Thus, time intervals of 1 month, 3 months, 6 months, or 1 year are not statistically significant for the diagnosis of sBBC and can be deferred to the follow-up schedule of the institute.

Therefore, the criteria for sBBC in this study were the demonstration of in situ carcinoma, stage 0-IIIa disease in bilateral breasts diagnosed within 12 months, and no evidence of local, regional, or distant metastasis.

### Potential Risk Factors for Primary sBBC

The incidence of UBC increased significantly over the years in this study, while that of sBBC remained constant [Figure 2], suggesting that the carcinogenesis of sBBC may be different from that of UBC [4], [5], [19]. There may be a more complicated host-carcinoma biological relationship in sBBC compared with UBC and even mBBC, in which there is a relatively long interval between two carcinomas, which is the reason why this study mainly focused on sBBC alone.

According to the results of multiple regression analysis and published studies [22]–[28], the risk factors for sBBC could be regarded as three aspects of the host-carcinoma biological relationship, including the tumor microenvironment, tumor cells, and genetic susceptibility.

We included accompanying benign disease in our analysis and unexpectedly found that the presence of sclerosing adenosis was the strongest independent risk factor for sBBC; this association has rarely been mentioned in the literature. Sclerosing adenosis is a subtype of adenosis associated with fibrocystic changes and the features of adenosis and stromal sclerosis. Ogura et al. suggested that cancer genuinely arising from sclerosing adenosis often had biological features of bilateral breast cancer and was negative for c-erbB-2 [20]. It was also suggested that careful examination of the contralateral breast should be recommended when ductal carcinoma in situ involving sclerosing adenosis is present in one of the breasts [21]. In this study, patients with carcinoma arising from or involved with sclerosing adenosis had an approximately 12-fold risk for developing sBBC compared with those without the presence of sclerosing adenosis. The proportion of patients with in situ carcinoma with or without microinvasion was astonishingly high at up to 64.3%, indicating a rather inert biological behavior. In addition, patients with sclerosing adenoma-associated sBBC had hormone receptor-positive disease in at least one breast, partially because the sclerosing adenosis was a lesion arising in the terminal duct-lobular unit.

Lobular carcinoma, including in situ and invasive disease, has a tendency to develop multicentric and bilateral carcinoma, and was demonstrated to be an independent risk factor for the development of sBBC in this study, which is widely supported by previous studies [8], [22], [23].

A family history of first-degree relatives with breast cancer may also play an important role in a subset of patients in developing bilateral carcinoma, as reported in several studies [9], [24], [25]. However, the prospective validation study did not confirm a direct relationship between family history and sBBC. A reasonable explanation for this discrepancy was the relatively close relationship between family history and genetic susceptibility; patients who carry a mutation in BRCA-1 or BRCA-2 may be associated with an increased risk of bilateral carcinoma [26]. In addition, genetic syndromes such as Cowden [27], Peutz-Jeghers [28], Li-Fraumeni [29], and Kindler syndrome [30] may also increase the incidence of BBC.

In addition to the above-mentioned risk factors, age at diagnosis was also confirmed as an independent risk factor for sBBC. However, patient age had a close relationship with many other factors, including tumor microenvironment, tumor cells, and genetic susceptibility. In a large population-based study, Hartmann et al. [4] found that the incidence of sBBC was at least twofold higher for women aged 80 years or older at diagnosis than those diagnosed when younger than age 40, indicated that the development of sBBC is more closely associated with the accumulation of environmental carcinogens and the gradual cancerization of the breast microenvironment than genetic determinants.

### Treatment and Survival of Patients with sBBC

The treatment and survival of patients with sBBC was not a major concern of this study. Partially because of the relative cautious attitude adopted by Chinese surgeons and patients in favor of systemic treatment and because the proportion of patients with sBBC who underwent bilateral mastectomy was extremely high at 90.1%, similar to other studies in the Chinese population [15], [26] but significantly higher than studies in other countries [7], [16]. A similar situation was found in the management of axilla in patients with sBBC.

This study supported similar survival between patients with sBBC and those with UBC, although the median follow-up period was relatively short [18]. However, for those with T_4_ or N_3_ disease in either breast, for whom it could be hard to distinguish metastatic and synchronous bilateral disease, the breast cancer-specific disease-free survival was extremely short: a mere 50%, with a median follow-up of 18 months.

## Conclusion

The presence of sclerosing adenosis, and lobular carcinoma component involvement are independent risk factors for sBBC. This study supports the hypothesis that the host-carcinoma biological relationship, especially for the tumor microenvironment, played a critical role in the carcinogenesis of sBBC.
